# From Sustainability to Regeneration: A Scoping Review of Nursing Practices in the Construction of Green and Healthy Hospitals

**DOI:** 10.3390/healthcare14172701

**Published:** 2026-08-24

**Authors:** Pablo Martín-Plaza, Jose Abad-Valle, Paloma Rodríguez-Gómez, Elena Arroyo-Bello, Estela Álvarez-Gómez, Beatriz González-Toledo, Belén González-Tejerina

**Affiliations:** 1 Fundación Jiménez Díaz School of Nursing, Universidad Autónoma de Madrid, 28040 Madrid, Spain; 2 Health Research Institute–Fundación Jiménez Díaz University Hospital, Universidad Autónoma de Madrid (IIS-FJD, UAM), 28040 Madrid, Spain

**Keywords:** sustainability, green hospitals, nursing, environmental health, carbon footprint, hospital waste management, nurse-sensitive environmental indicators, regenerative healthcare

## Abstract

**Background/Objectives:** The healthcare sector accounts for an estimated 1–5% of the global environmental footprint, and hospitals concentrate a large share of that impact. As the largest professional group, nurses are pivotal to the transition toward green and regenerative hospitals. This review aimed to map the sustainable nursing practices implemented in hospitals internationally and to characterise the contribution of nursing to reducing the environmental footprint of care. **Methods:** A scoping review was conducted following the Joanna Briggs Institute methodology and reported in accordance with the Preferred Reporting Items for Systematic Reviews and Meta-Analyses extension for Scoping Reviews (PRISMA-ScR). PubMed, Scopus, ScienceDirect, SpringerLink, SciELO and Dialnet were searched for studies published between 2021 and 2026, complemented by grey and institutional literature, citation searching and hand-searching. Records were screened in duplicate against predefined eligibility criteria, and the evidence was charted into six thematic categories aligned with the review objectives. **Results:** Twenty-five sources were included—11 reviews and 14 primary studies, most of them descriptive. Waste management emerged as the domain most sensitive to nursing action, whereas energy and water use were only minimally nurse-sensitive. Education, leadership and nurse engagement were the main enablers; insufficient training, resistance to change and limited investment were the recurrent barriers. Only one study explicitly addressed the regenerative transition, from a governance perspective. **Conclusions:** Embedding sustainable practices in nursing can reduce the environmental impact of hospitals while preserving quality of care. Nurse-sensitive environmental indicators and the integration of sustainability competencies into curricula and hospital governance are key levers for this transition, whose regenerative dimension remains incipient and requires primary, comparative research.

## 1. Introduction

Climate change and environmental degradation are among the most pressing challenges of the twenty-first century, and the healthcare sector is simultaneously a victim and a contributor. Several global assessments estimate that health systems are responsible for between 1% and 5% of the total environmental footprint of the planet, with impacts that extend across at least seven domains, including greenhouse-gas emissions, particulate matter, air pollutants, malaria risk and reactive nitrogen and water use [1]. Converging sector-wide analyses estimate that healthcare’s climate footprint is equivalent to approximately 4.4% of global net greenhouse-gas emissions—if it were a country, the sector would be the fifth-largest emitter on the planet [2]—and successive reports of the Lancet Countdown document a growing health burden of climate change to which health systems both respond and contribute [3]. Hospitals concentrate a disproportionate share of this footprint because they operate continuously, consume large amounts of energy and water, and generate substantial volumes of hazardous and non-hazardous waste.

In response, the international community has developed frameworks to reorient health systems toward environmental responsibility. The Global Green and Healthy Hospitals agenda, promoted by the non-governmental organisation Health Care Without Harm, articulates this ambition through ten interconnected goals applicable to both health systems and individual hospitals: leadership, chemicals, waste, energy, water, transportation, food, pharmaceuticals, buildings and purchasing [4]. In England (the United Kingdom), for example, the National Health Service (NHS) net-zero roadmap has set explicit decarbonisation targets through reduced travel, renewable energy, efficient use of medicines and supply-chain engagement [5]. Increasingly, this aspiration is moving beyond merely reducing consumption toward the notion of regeneration—hospitals and organisations capable of generating resources and actively contributing to the restoration of the ecosystems that sustain them, rather than only minimising harm.

Nurses are central to this transition. Professional bodies have formalised this responsibility: the International Council of Nurses calls on nurses and their associations to engage in sustainable practice, correct waste management and climate-informed models of care [6], and conceptual work in nursing science has characterised sustainability—through the attributes of ecology, environment, future orientation, globalism, holism and maintenance—as an emerging component of the discipline [7]. They constitute the largest professional group within the health workforce and maintain the closest and most continuous contact with patients, materials and clinical processes; consequently, their decisions have a direct and cumulative effect on resource use and waste generation. Despite a growing number of initiatives, the evidence consistently points to a lack of information, training and environmental awareness among health professionals, together with a shortage of indicators and protocols that safeguard sustainability in the workplace; a decade after the concept was first formalised for nursing, its integration into practice, education and policy is still described as incomplete [8]. Reorienting nursing practice toward more sustainable models would therefore improve resource use and substantially reduce the emissions generated by the sector, yielding a fairer, more effective and more environmentally respectful health system.

This review builds on a body of prior work. Sector-wide analyses have quantified the climate footprint of healthcare and identified its principal sources [2], earlier syntheses have mapped sustainable supply-chain management practices in hospitals [9] and waste-reduction strategies in clinical settings [10], and nursing scholarship has examined sustainability at the conceptual level for more than a decade [7,8]. What remained unmapped was the specific, practice-level contribution of nursing to green-hospital strategies in the most recent period, which is the gap this review addresses.

Against this background, the aim of this review was to map the sustainable nursing practices currently implemented in hospitals internationally, without geographic restriction—paying particular attention to the transferability of the findings to the European context in which the review team practises—and to characterise their contribution to reducing the environmental footprint of care. The specific objectives were: (i) to identify energy-efficiency strategies that favour sustainability in nursing practice; (ii) to examine methods of hospital waste management and their relationship with nursing activities; (iii) to evaluate the carbon footprint associated with nursing activities and proposals for its reduction; (iv) to investigate the use of ecological materials in nursing practice and their impact on hospital sustainability; (v) to appraise the social and health-related values of sustainable measures applied by nurses; and (vi) to identify and describe innovative projects related to the transition toward regenerative hospitals and the role of nursing in their implementation.

The temporal scope of the review (2021–2026) was chosen to capture the evidence generated after the major policy milestones that reconfigured this field—most notably the launch of national net-zero strategies for health systems [5] and the consolidation of the Global Green and Healthy Hospitals agenda [4]—and to map the current, rather than historical, nursing practice; seminal works published before 2021 were retained only as contextual references. Within this scope, the review is international by design, and the European context is treated as the primary setting for the interpretation and transfer of the findings rather than as a geographic filter.

## 2. Materials and Methods

### 2.1. Study Design

**Figure 1:**
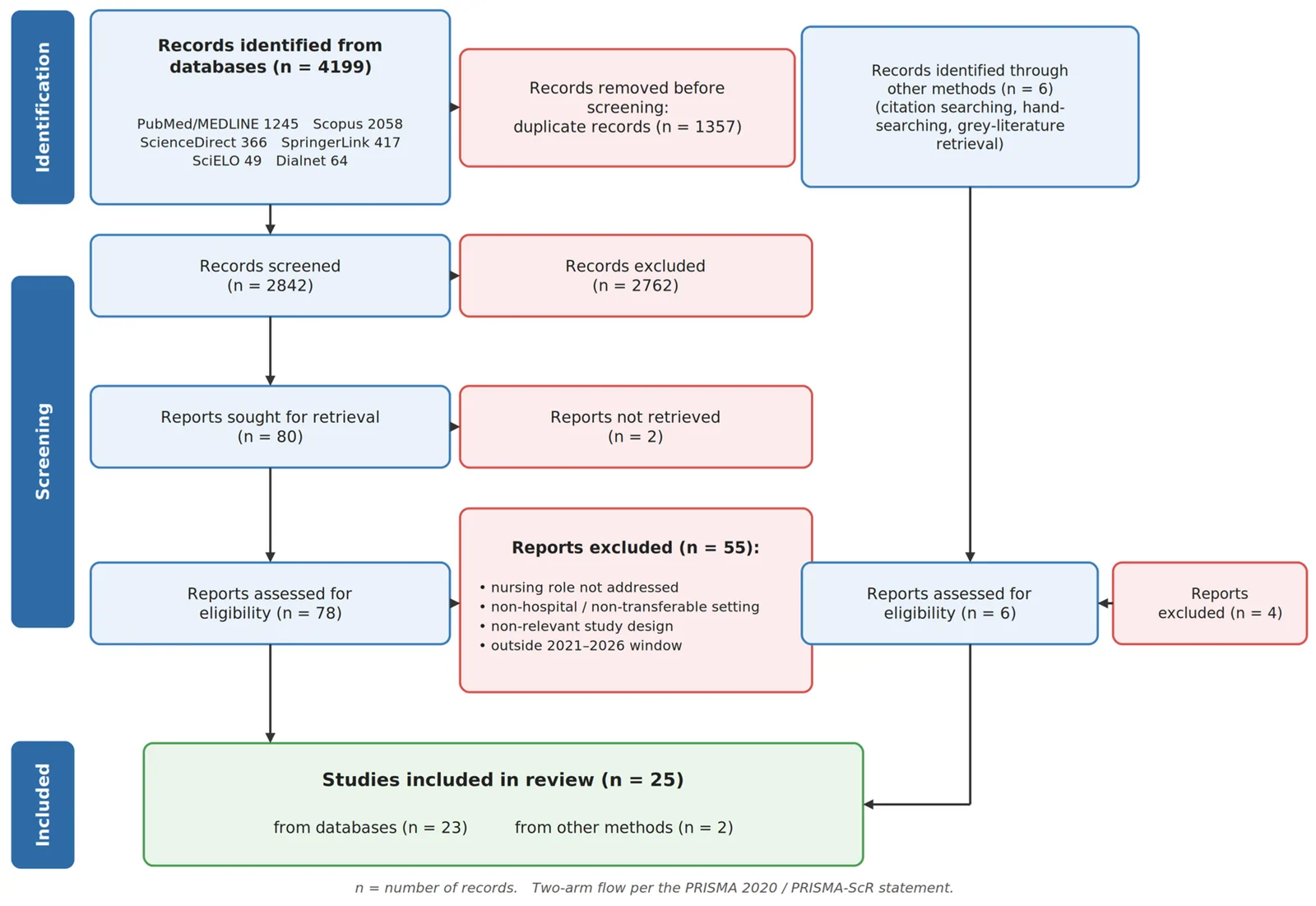
PRISMA-ScR two-arm flow diagram of the study-selection process (records identified through databases and registers, and through other methods). PRISMA-ScR, Preferred Reporting Items for Systematic Reviews and Meta-Analyses extension for Scoping Reviews.

A scoping review of the literature was conducted to map the nature, extent and range of the available evidence on environmental-sustainability strategies in nursing practice within the hospital setting. The review followed the Joanna Briggs Institute (JBI) methodology for scoping reviews and the framework of Arksey and O’Malley and was reported in accordance with the Preferred Reporting Items for Systematic Reviews and Meta-Analyses extension for Scoping Reviews (PRISMA-ScR); a PRISMA-ScR flow diagram is provided (Figure 1). A scoping-review design was chosen, rather than a systematic review of effectiveness, because the aim was to map a heterogeneous body of evidence (qualitative research, descriptive observational studies, case reports, narrative and systematic reviews, and official good-practice reports) and to integrate not only effectiveness findings but also experiences, management models and good-practice guidance, while preserving methodological transparency and reproducibility.

To structure the review, the Population–Concept–Context (PCC) framework recommended by the JBI for scoping reviews was used. Population (P): nursing professionals—and, where relevant, other health professionals—working in hospitals. Concept (C): the implementation of sustainable practices related to energy efficiency, waste management, the use of ecological materials, hospital sustainability, carbon-footprint reduction and the transition toward regenerative hospitals, together with their contribution to reducing resource consumption and the environmental footprint of care. Context (C): the hospital setting in any country or region, without geographic restriction; particular attention was paid to the transferability of the findings to the European context, in which the review team practises. Consistent with scoping-review methodology, no comparator was defined, since the objective was to map the available evidence rather than to compare the effectiveness of interventions.

*Protocol and registration*. The review protocol was registered retrospectively in the Open Science Framework (OSF; registration DOI: https://doi.org/10.17605/OSF.IO/36KAG). The protocol was designed and the search strategy developed between September 2024 and May 2026, with the period corresponding to the full conduct of the review; because OSF date-stamps the actual deposit, the retrospective nature of the registration is acknowledged explicitly as a transparency measure and is also reported as a limitation. Screening and data charting were carried out independently and in duplicate by two reviewers (P.M.-P. and J.A.-V.), with disagreements resolved by consensus. Because the registration was retrospective, all deviations from the initially drafted protocol are itemised explicitly: (i) the expansion of the review team from three to seven members in July 2025, during the conduct phase; (ii) the clarification of the geographic scope—the initially drafted eligibility wording referred to hospitals located in European territory while simultaneously admitting transferable examples from other continents, and in execution the criterion was applied as an international scope with particular attention to European transferability, so both the eligibility wording (Section 2.3) and the search strategies (removal of the geographic block from PubMed and Scopus; Section 2.2 and Supplementary Material S1) have been aligned with the procedure actually applied; and (iii) the adaptation of the ScienceDirect strategy to the title, abstract and keywords fields after an initial title-only query returned no records (Section 2.2). The research questions, the specific objectives and the dual independent screening and charting procedures were not modified after registration. All updates are documented transparently in the editable component of the OSF project (https://osf.io/8q2pk, accessed on 19 August 2026), while the registration snapshot itself remains immutable.

### 2.2. Data Sources and Search Strategy

The bibliographic search was performed in six bibliographic databases selected to cover the multidisciplinary scientific literature (biomedical, social, economic and environmental): PubMed/MEDLINE, Scopus, ScienceDirect, SpringerLink, SciELO and Dialnet. Scopus was added to ensure broad multidisciplinary coverage and to meet the journal’s expectation of a major international database. This was complemented by a targeted search of the institutional grey literature of recognised authority (e.g., the World Health Organization, the Spanish Ministry of Health, Health Care Without Harm and national health-service reports). The Universidad Autónoma de Madrid library catalogue was used as a retrieval tool for grey and institutional literature rather than as a counted bibliographic database, and its results are reported within the other-methods arm of the flow diagram. Citation searching and reference-list hand-searching of key nursing and sustainability journals were also undertaken. The search strategy combined controlled vocabulary (Medical Subject Headings [MeSH] and Descriptors in Health Sciences [DeCS]) and free-text terms relating to nursing, hospital sustainability, energy efficiency, waste management, ecological materials, carbon footprint and regenerative hospitals, using Boolean operators (AND, OR, NOT) and truncation. The Boolean strings were developed, calibrated and translated across databases using the MCL Search Calibrator (Mnemonics, Concepts and Links), version 1.0.0, which maps concepts to controlled vocabularies (MeSH and Emtree), generates database-specific syntax for PubMed, CINAHL (Cumulative Index to Nursing and Allied Health Literature) and Scopus, and audits the strategy following the Peer Review of Electronic Search Strategies (PRESS) checklist; for the remaining databases the strings were adapted manually to their own syntax. An earlier version of the PubMed and Scopus strategies included a geographic limiter restricting results to Europe; this block was removed because it was inconsistent with the eligibility criteria, which expressly admit transferable examples of good practice from other regions, and the strategies are therefore reported and run without geographic restriction (this protocol deviation is documented in Supplementary Material S1). Removing the geographic limiter increased the sensitivity of the search at the expense of screening workload rather than of precision: the additional records retrieved were adjudicated against the unchanged eligibility criteria, applied independently and in duplicate at both the title/abstract and full-text stages, so that geographic and contextual relevance was decided—and can be audited—at the screening stage rather than silently at the search stage. In ScienceDirect, the platform searches full text by default and caps Boolean complexity, so the multi-concept strategy was applied to the title, abstract and keywords fields and captured across successive result pages, returning 366 records; an earlier title-only query had returned no records and underestimated the coverage of the database. Elsevier journal content is also comprehensively indexed in Scopus, which was searched. SpringerLink was restricted by content type (Article), discipline (Medicine and Public Health) and publication year (2021–2026). SciELO was limited to 2021–2026 and to English- and Spanish-language records, returning 49 records. In Dialnet, four complementary term combinations returned 4, 7, 45 and 31 records (87 in total); after removing records retrieved by more than one combination, 64 unique records were retained. These database-specific adaptations are documented in Supplementary Material S1. The last search was run on 28 May 2026. To ensure reproducibility, the complete and literal Boolean string used in PubMed/MEDLINE is reported below, and the syntactic equivalents for the remaining databases are provided as Supplementary Material; the number of records retrieved from each individual source is reported in Section 3.1 (see also Figure 1). Results were limited to publications in English and Spanish from 2021 to 2026. The detailed sources and search terms are summarised in Table 1.

*Literal PubMed/MEDLINE search string (run on 28 May 2026):* (“Nurses”[Mesh] OR nurs*[tiab] OR “nursing staff”[tiab] OR “nursing personnel”[tiab]) AND (“Hospitals”[Mesh] OR hospital*[tiab] OR “health facilit*”[tiab] OR “healthcare facilit*”[tiab] OR “medical cent*”[tiab]) AND (“Sustainable Development”[Mesh] OR “Conservation of Natural Resources”[Mesh] OR “Carbon Footprint”[Mesh] OR sustainab*[tiab] OR “green hospital*”[tiab] OR “green health*”[tiab] OR “energy efficien*”[tiab] OR “energy conservat*”[tiab] OR “waste management”[tiab] OR “medical waste”[tiab] OR “healthcare waste”[tiab] OR “eco-friendly material*”[tiab] OR “sustainable procurement”[tiab] OR “green material*”[tiab] OR “carbon footprint*”[tiab] OR “greenhouse gas*”[tiab] OR decarboni*[tiab] OR “net zero”[tiab] OR (regenerative[tiab] AND hospital*[tiab]) OR “regenerative health*”[tiab]) [The geographic “Europe” block has been removed from this string; see Section 2.2 and Supplementary Material S1].

### 2.3. Eligibility Criteria

Inclusion criteria were: (1) studies conducted in the hospital setting in any country or region, provided that the practices described were transferable to the review objectives, with particular attention to the European context (see Section 2.1 for the clarification of the initially drafted wording); (2) explicit description of the direct or indirect participation of nursing professionals in the development or implementation of sustainable strategies; (3) original studies (quantitative or qualitative research, case studies, and systematic or narrative reviews with methodological rigour); (4) content related to at least one of the specific objectives of the review (energy efficiency, waste management, carbon-footprint reduction, use of ecological materials, promotion of environmental health, or initiatives toward regenerative hospitals); and (5) publications between 2021 and 2026, in English or Spanish.

Exclusion criteria were: (1) studies that did not explicitly address the role of nursing or the impact of sustainable practices on nursing activity; (2) non-peer-reviewed publication types of purely opinion-based nature (letters to the editor, personal opinions and blogs); and (3) duplicate documents. In keeping with scoping-review methodology, studies were not excluded on the basis of a formal methodological-quality (critical appraisal) score; instead, the methodological characteristics of the included sources were charted descriptively (see Section 2.5).

### 2.4. Screening and Selection Procedure

The selection process was conducted in successive phases following the PRISMA-ScR scheme. In the identification phase, all search results were exported to a reference manager (Mendeley) and organised according to the source and the search terms employed. After duplicate and inaccessible records were removed, two reviewers (P.M.-P. and J.A.-V.) independently screened titles and abstracts to discard studies that did not meet the inclusion criteria or fell outside the scope of sustainability in nursing practice. Potentially relevant articles were then read in full by both reviewers to verify their adequacy regarding the review objectives, and only those that fully met the inclusion criteria and provided substantive information were retained. In addition to the database searches, the reference lists of all included studies were hand-searched (citation searching/snowballing), key nursing and sustainability journals were scanned, and the Universidad Autónoma de Madrid library catalogue and the institutional grey-literature sources were consulted to identify further relevant or contextual sources. These supplementary searches constituted a second identification stream (other methods), whose records were de-duplicated against the database results and screened against the same eligibility criteria. Discrepancies at any stage were resolved by discussion until consensus was reached. Consistent with the PRISMA-ScR extension, the complete flow of records is presented as a two-arm diagram, distinguishing records identified through databases and registers from those identified through other methods (Figure 1).

### 2.5. Data Extraction and Synthesis

A data-charting template was designed to organise the information in a clear and coherent manner, capturing: the bibliographic reference (authors, year, title, source); the objective or main purpose of the document; the methodological design and sample size, where applicable; the country or setting and the type of hospital or unit; the principal findings regarding sustainable practices led by or linked to nursing (energy efficiency, waste, carbon footprint, etc.); and the authors’ conclusions and recommendations. The data were charted by one reviewer and verified by a second, and the results were collated through a descriptive narrative analysis that grouped findings into thematic categories aligned with the review objectives. Consistent with scoping-review methodology, no formal critical-appraisal score was used to determine the inclusion or exclusion of studies. Instead, each included source was classified by type of evidence (systematic, scoping, narrative, expert or historical review; quantitative observational or quasi-experimental study; qualitative study; mixed-methods study; Delphi consensus study; descriptive institutional study; historiographic study), and this classification was used to structure the narrative synthesis: findings supported by primary studies reporting measured outcomes were distinguished from those derived from reviews or from descriptive and institutional sources, and all quantitative figures cited in the Results are attributed to their original source and treated as context-specific estimates rather than pooled effects. A formal critical appraisal with design-specific instruments (e.g., the JBI critical-appraisal tools) was considered but not adopted, consistent with JBI guidance and with PRISMA-ScR item 12, which treats appraisal as optional for scoping reviews; the implications of this decision for the weight of the mapped evidence are examined critically in Section 4.7.

### 2.6. Ethical Considerations

Because this is a review of secondary studies, approval by a research ethics committee was not required. Nonetheless, it was confirmed, as far as possible, that the included studies had complied with ethical and legal criteria at source (informed consent, confidentiality and respect for local and international research regulations). The principles of academic integrity and appropriate use of information were respected, avoiding plagiarism and correctly citing all sources to guarantee transparency and scientific accountability.

### 2.7. Use of Generative Artificial Intelligence

**Figure 2:**
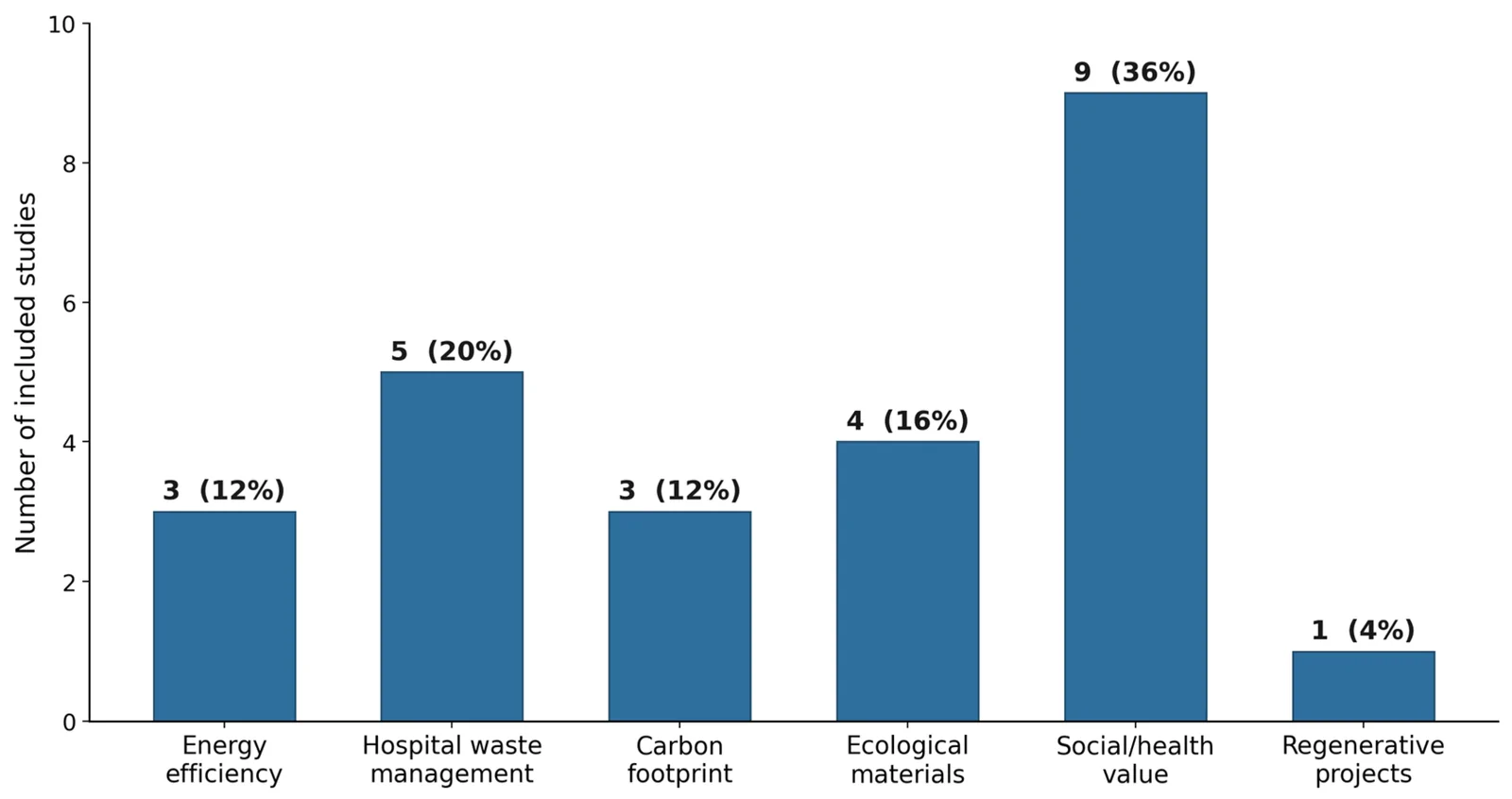
Distribution of the 25 included studies across the six thematic categories.

During the preparation of this manuscript, the authors used two generative artificial intelligence tools—the large language models Claude Opus 4 and Claude Fable 5, developed by Anthropic (San Francisco, CA, USA) and accessed through the Claude web interface (https://claude.ai, accessed on 19 August 2026). Under the direct supervision of the authors, these tools were used exclusively for the following support tasks: (i) language editing of the English text drafted by the authors, to improve grammar, clarity and readability; (ii) formatting of the manuscript and tables in accordance with the journal’s Microsoft Word template; (iii) technical preparation of the figures (the PRISMA-ScR flow diagram in Figure 1 and the thematic distribution chart in Figure 2) from data and content provided by the authors; and (iv) formatting of the reference list according to the journal’s bibliographic style. These tools were not used to conceive the study, design the search strategy, search the literature, select studies, chart or analyse the data, interpret the findings, or generate scientific content, hypotheses or conclusions; all of these tasks were performed entirely by the authors. All output produced with the assistance of these tools was reviewed, verified against the original sources, and edited by the authors, who take full responsibility for the content of this publication.

## 3. Results

### 3.1. Study Selection

The calibrated search, run without a geographic limiter in the database strings (see Section 2.2), identified 4199 records across the six databases (PubMed/MEDLINE, 1245; Scopus, 2058; ScienceDirect, 366; SpringerLink, 417; SciELO, 49; and Dialnet, approximately 64 unique). In ScienceDirect the strategy was applied to the title, abstract and keywords fields rather than to the title alone, which returned 366 records; the earlier title-only query had returned none and underestimated the coverage of the database. After 1357 duplicate records had been removed, 2842 records were screened by title and abstract, of which 2762 were excluded. Eighty reports were sought for retrieval; two could not be obtained, and the remaining 78 were assessed in full against the eligibility criteria, of which 55 were excluded (mainly because they did not focus on nursing practices, addressed a non-hospital setting or described strictly context-bound circumstances without nursing practices transferable to the review objectives (the operational application of criterion 1), used a non-relevant study design, or fell outside the 2021–2026 publication window). The other-methods arm—citation searching, hand-searching of key journals, and grey-literature retrieval via the Universidad Autónoma de Madrid catalogue and institutional sources—identified six further reports, of which four were excluded and two were included. In total, 25 studies were included in the synthesis (23 through the database arm and two through the other-methods arm). The complete selection process is shown in Figure 1.

Table 2 summarises the 25 included studies, indicating the design, the setting or population, and the key findings concerning the implementation of sustainable nursing practices in hospitals for each one. The evidence was grouped into six thematic categories defined by the review objectives, whose distribution is shown in Figure 2.

In terms of the type of evidence, the 25 included sources comprised 11 reviews (three systematic reviews [11,12,13], two scoping reviews [14,15], four narrative reviews [16,17,18,19], one expert review [20] and one historical narrative review [21]) and 14 primary studies (six quantitative observational or quasi-experimental studies [22,23,24,25,26,27], four qualitative studies [28,29,30,31], one mixed-methods study [32], one Delphi consensus study [33], one descriptive institutional study [34] and one historiographic study [35]). Geographically, seven sources reported European settings [13,24,25,29,30,31,33], one was conducted in Türkiye [27], five in the Americas [16,22,28,32,34], one in Africa [23] and one in Oceania [26], while ten adopted a global or multi-country perspective or did not specify a single national setting [11,12,14,15,17,18,19,20,21,35]. The retrieved evidence is therefore international rather than predominantly European, and reviews and descriptive designs outnumber comparative primary studies; both features are weighed explicitly in the Discussion when interpreting the strength of individual findings.

**Table 2:** Characteristics and key findings of the 25 included studies.

Study (Author, Year)	Design	Thematic Category	Setting/Population	Key Findings
Schenk & Johnson, 2022 [28]	Qualitative descriptive	Energy efficiency	Environmental-stewardship nurse leaders, USA	Defines nurse-sensitive environmental indicators (NSEIs); waste is highly nurse-sensitive, food/chemicals/transport moderately, and energy/water only minimally sensitive.
Schenk et al., 2023 [16]	Narrative review	Energy efficiency	US healthcare-waste system	Proposes a waste-optimisation plan (appropriate segregation, reduction in single-use products, sustainable practice) and highlights the nurses’ central role and the energy cost of inefficient resource use.
Jester, 2024 [20]	Expert review	Energy efficiency	Orthopaedic and trauma nursing	Advocates embedding sustainability in orthopaedic and trauma nursing through waste reduction, renewable energy, waste-heat recovery and reduced procedure-related emissions and transport.
Chatzipavlou et al., 2024 [14]	Scoping review	Hospital waste management	Hospital food-service systems	Around 30% of food served to patients is wasted (dietary restrictions, quality, taste, inefficient management)—a major source of environmental impact for which no proposal has yet achieved effective control.
Kenny & Priyadarshini, 2021 [11]	Systematic review	Hospital waste management	Global healthcare-waste systems	Heavy reliance on incineration and landfilling with damaging environmental and public-health effects; greener alternatives remain limited by cost, access and feasibility.
Gallegos-Pasco & Pineda-Chaiña, 2022 [22]	Case study with controls	Hospital waste management	Manuel Núñez Butrón Hospital, Puno, Peru	Proposes an ISO 14001 environmental-management system for water, air, soil and waste to significantly reduce the hospital’s environmental impact.
Mostepaniuk et al., 2023 [12]	Systematic review	Hospital waste management	Healthcare organisations (global)	Effective practices include improved management and leadership, clinician engagement, virtual health communication, green supply-chain management, rational resource use and waste management.
Moreno Sánchez, 2022 [17]	Narrative review	Carbon footprint	Populations affected by environmental change (global)	Establishes a relationship between environmental change and health risk; reports that ~23% of deaths worldwide (and ~24% of the global burden of disease) are environment-related and stresses environmental factors as a clinical risk.
Pionce-Acosta & Mieles-García, 2023 [32]	Descriptive, mixed methods	Carbon footprint	Hospital in Jipijapa, Ecuador	Identifies deficiencies in waste management (inadequate containers, deficient internal transport) that affect air, soil and water, and on which nurses have decisive influence.
Hossny, 2022 [23]	Descriptive observational (work sampling)	Carbon footprint	Inpatient units, university hospital, Egypt	Unclassified and personal activities consume a large share of nursing time (44.1%, 41.6% and 55.2% in ICU, medical and surgical units), reducing efficiency and sustainability.
Vanderwee et al., 2025 [24]	Cross-sectional study	Ecological materials	Hospitals in Belgium	Develops a practical method to choose between single-use and reusable materials based on environmental impact, cost, safety and efficiency; identifies five widely used items with sustainable alternatives.
Wu & Cerceo, 2021 [18]	Narrative review	Ecological materials	Operating rooms/surgical areas	Operating rooms generate up to 30% of hospital waste; proposes sustainability teams, recycling, appropriate segregation, sustainable anaesthetic gases, and energy and water reduction.
Masud et al., 2024 [19]	Narrative review and hospital-system experience	Ecological materials	Intensive care units (global)	Proposes a three-step pathway to a “Green ICU”: quantify the carbon footprint, build alliances, and implement initiatives with active participation of ICU staff.
Palacios Vintimilla & Erazo-Álvarez, 2021 [34]	Descriptive study	Social/health value	Homero Castanier Crespo Hospital, Azogues, Ecuador	Proposes an environmental social-responsibility plan to reduce the negative impact of hospital activity, underlining the lack of climate awareness among staff.
Galvão et al., 2023 [15]	Scoping review	Social/health value	Hospitals in several countries	Identifies water and energy consumption, waste treatment and pollutant emissions as fundamental indicators for reducing hospitals’ environmental impact.
Rodríguez-López et al., 2024 [21]	Historical narrative review	Social/health value	Nursing, environment and sustainability (global)	Compiles seven key milestones from Nightingale’s environmental theory to sustainable initiatives in Latin America, stressing the need to train nurses in green policies.
Luque Alcaraz et al., 2021 [35]	Historiographic study	Social/health value	Nursing care and environmental sustainability over time	Shows that nursing care has always been linked to environmental care; highlights initiatives such as hospital gardens and the role of the care environment in healing.
van Schie, 2024 [13]	Systematic review	Regenerative projects	European hospitals	Four governance factors influence implementation—knowledge, management involvement, professional commitment and technology use—currently acting mainly as barriers.
Lucchini et al., 2026 [25]	Retrospective evaluation	Hospital waste management	ICU, Italy	Compared single-use versus reusable products across six respiratory procedures in an ICU, quantifying the plastic-waste burden of each option and indicating scope for nurse-led substitution to cut disposable waste.
Bartoli et al., 2025 [29]	Qualitative (content analysis)	Social/health value	27 ICU nurses, Italy	ICU nurses recognised intensive care as a major source of hospital emissions and waste and were willing to act but struggled to define sustainability; the authors call for structured education and support from scientific societies.
Figura et al., 2026 [30]	Qualitative (lexicometric)	Social/health value	29 critical-care nurses, Italy	A lexicometric analysis of nurses’ narratives mapped how critical-care nurses construct the meaning of environmental sustainability, showing that professional culture and clinical context shape sustainable practice.
Feely et al., 2025 [26]	Before–after study	Ecological materials	ICU, Australia	Replacing single-use disposable pads with reusable linen did not increase pressure-injury rates, eliminated about 496 kg of waste per year and achieved high staff satisfaction.
López-Medina et al., 2022 [31]	Cohort (content analysis)	Social/health value	Nursing students, Spain	Students valued sustainability training but were concerned about excessive use of disposable materials, poor waste segregation and limited capacity to act, and demanded more low-impact-care content.
Batino et al., 2026 [33]	Delphi study	Social/health value	Health professionals, Italy	Surveyed the knowledge and perception of climate change among health professionals to identify priorities for structured environmental-sustainability education.
Çolak & Karakaya, 2026 [27]	Cross-sectional study	Social/health value	Paediatric-clinic nurses, Türkiye	Higher environmental literacy among nurses in paediatric clinics was associated with greater climate-change awareness, supporting literacy-building initiatives.

ICU, intensive care unit; ISO, International Organization for Standardization; NSEI, nurse-sensitive environmental indicator.

The findings are described below according to the specific objectives of the review.

### 3.2. Energy-Efficiency Strategies in Nursing Practice

Three studies addressed energy efficiency and its relationship with nursing practice. A recurrent finding was that the domain in which nurses exert the greatest influence is waste generation, whereas energy and water consumption are less directly modulated by their actions. Schenk and Johnson [28] formalised this insight through the concept of nurse-sensitive environmental indicators (NSEIs), identifying waste management as highly sensitive to nursing intervention and energy and water use as only minimally sensitive. In a complementary study, Schenk et al. [16] documented the considerable impact of inefficient management of both resources and energy systems in hospitals. In orthopaedic and trauma surgery, Jester [20] highlighted material and energy consumption together with patient and staff transport and the classification of paper-based medical records.

### 3.3. Hospital Waste Management and Nursing Activities

Five studies examined hospital waste management in relation to nursing activities. Beyond the seminal supply-chain perspective of Duque-Uribe et al. [9], who identified up to twelve categories of practice capable of making hospitals greener (including efficient supplier management and responsible water use), the included studies focused on substituting current disposal methods with more sustainable alternatives. Chatzipavlou et al. [14] reported that approximately 30% of the food served to patients ends up as waste—mainly because of dietary restrictions, food quality and taste, and the absence of an efficient management system—contributing substantially to waste generation and environmental pollution, with no existing proposal having achieved effective control. The analysis by Kenny and Priyadarshini [11] showed that although greener alternatives such as reuse and recycling exist, their implementation remains limited by economic and logistical barriers. Initiatives such as the International Organization for Standardization (ISO) 14001 environmental-management system proposed by Gallegos-Pasco and Pineda-Chaiña [22] in Peru illustrate concrete actions under way, while Mostepaniuk et al. [12] underlined the importance of integrating environmental, social and economic measures—green technologies, reduced energy consumption, digital health and improved working conditions—into hospital management. In the same vein, Lucchini et al. [25] retrospectively compared single-use and reusable products across six respiratory procedures in an intensive-care unit, quantifying the plastic-waste burden of each option and showing the scope for nurse-led substitution to reduce disposable waste.

### 3.4. Carbon Footprint of Nursing Activities and Strategies for Its Reduction

Three studies investigated the carbon footprint associated with nursing activities and proposals for its reduction. Moreno Sánchez [17] reported that approximately 23% of deaths worldwide (and around 24% of the global burden of disease) are attributable to environmental factors, underscoring the importance of treating them as a clinical risk factor. Pionce-Acosta and Mieles-García [32] identified deficiencies in waste-management methods—such as inadequate containers and deficient internal transport—on which nurses have decisive influence and represent a key element for improvement. At the macro level, the broader literature situates the health sector’s contribution at 1–5% of the global environmental impact across seven pollutant factors [1]; in Spain, the Strategic Plan for Health and the Environment (PESMA) adds chemical exposure to these factors and proposes the One Health approach, integrating human, animal and environmental health [36]. The efficient use of nursing time also emerged as a lever: Hossny [23] observed that unclassified and non-essential activities consumed the largest share of nursing time—44.1%, 41.6% and 55.2% in intensive-care, inpatient and surgical units, respectively—negatively affecting the efficiency and sustainability of the profession.

### 3.5. Ecological Materials in Nursing Practice

Four studies explored the use of sustainable materials in nursing practice and their environmental impact. Vanderwee et al. [24] developed an effective, practical method to help hospitals choose more sustainable alternatives, based on an inventory of disposable resources, prioritisation according to cost, consumption and reusability, and life-cycle analysis to evaluate environmental impact, safety, cost-effectiveness and efficiency; applying this method, they identified five widely used items—kidney dishes, vaginal specula, thermometer sleeves, vessel sealers and patient blankets—with more sustainable alternatives. Concrete unit-level measures were also reported. Wu and Cerceo [18] noted that operating rooms generate up to 30% of hospital waste owing to their intensive use of disposable materials, and proposed sustainability teams, recycling and reuse programmes, appropriate waste segregation, minimisation of unnecessary devices and packaging, reduced energy and water consumption, the transition to more sustainable anaesthetic gases, staff education and the donation of unused supplies. For intensive care units (ICUs), Masud et al. [19] described a three-step pathway toward a “Green ICU”: quantifying the carbon footprint of these resource-intensive units, creating initiatives to reduce emissions and waste, and implementing a green-ICU system with the active participation of staff. The broader literature similarly identifies hospital laundries and the inefficient use of single-use resources as priority areas for waste reduction, again stressing the central role of nursing [10]. Extending this materials focus to bedside textiles, Feely et al. [26] conducted a before–after study in an intensive-care unit and found that replacing single-use disposable pads with reusable linen did not increase pressure-injury rates while eliminating roughly half a tonne of waste per year and earning high staff satisfaction.

### 3.6. Social and Health-Related Value of Sustainable Nursing Measures

Nine studies appraised the socio-health value of sustainable measures applied by nursing. Both Palacios Vintimilla and Erazo-Álvarez [34] and Galvão et al. [15] observed a lack of climate awareness among health staff, resulting in poor waste handling and increased greenhouse-gas emissions from poorly optimised practices, with consequent water pollution, odour problems and deterioration of air quality that worsen population health; both underscored the importance of nursing involvement and the need for training and awareness in environmental matters. From a historical perspective, Luque Alcaraz et al. [35] described how the nursing profession has always been intrinsically linked to environmental care, with Florence Nightingale highlighting light and ventilation as part of the healing process, while Rodríguez-López et al. [21] traced seven key milestones in the relationship between nursing and sustainability—from Nightingale’s environmental theory to the integration of sustainable initiatives in Latin America—reinforcing the need to train nurses in green policies and environmental awareness. Two recent qualitative studies of intensive-care nurses reinforced this social dimension: Bartoli et al. [29] found that critical-care nurses recognise the environmental footprint of intensive care and are willing to act but lack a clear definition of sustainability and call for structured education, while Figura et al. [30] used a lexicometric analysis of nurses’ narratives to show how professional culture and clinical context shape the meaning that nurses give to sustainable practice. The educational and attitudinal dimension was further documented across settings: in a cohort of Spanish nursing students, López-Medina et al. [31] reported concerns about the excessive use of disposable materials and the poor segregation of waste, together with a demand for more training in low-impact care; Batino et al. [33], in a Delphi study of Italian health professionals, characterised the knowledge and perception of climate change as a basis for targeted education; and Çolak and Karakaya [27] showed, among nurses in paediatric clinics, that strengthening environmental literacy is associated with greater climate-change awareness.

### 3.7. Innovative Projects Toward Regenerative Hospitals

One included study focused directly on the governance of the transition toward more sustainable hospitals, complemented by an institutional example of implementation. van Schie [13] identified the governance factors that influence the implementation of ecological dynamics in European hospitals—knowledge of sustainability, promotion of green policies, professional commitment and the use of technology—and described the limitations that currently impede these initiatives. These factors operate within the framework of the Global Green and Healthy Hospitals agenda, whose ten goals provide a comprehensive roadmap for hospital sustainability [4], and of national decarbonisation strategies such as the NHS net-zero roadmap [5]. In Spain, the MAS+ (Medio Ambiente y Salud, Environment and Health) programme currently implemented at the Fundación Jiménez Díaz University Hospital exemplifies this transition: starting from the premise that environmental pollution worsens population health and thereby contradicts the very mission of the health sector, the programme introduced training workshops, sustainable prescribing (reducing carbon emissions associated with inhalers and anaesthetic gases such as nitrous oxide by 13.5%), a 16.4% reduction in paper use, a 17% increase in video consultations, bicycle parking and electric-vehicle charging points, and improved waste management and sustainable purchasing, achieving an overall 14.07% reduction in the hospital’s total emissions [37]. It should be noted, however, that no included study evaluated a regenerative intervention as such: the only source that explicitly addresses the transition toward regenerative hospitals does so from a governance perspective [13], and the MAS+ programme [37] is an institutional decarbonisation example rather than a regenerative project in the strict sense. The evidence charted under this objective is therefore best characterised as governance- and sustainability-oriented groundwork for a regenerative model, not as evidence of regeneration itself.

## 4. Discussion

The detailed review of the 25 included studies confirms and extends the knowledge that frames the environmental challenge of the hospital sector. Two cross-cutting messages emerge consistently: first, that hospitals concentrate a substantial and avoidable environmental burden; and second, that nursing—by virtue of its size and its proximity to patients and materials—is uniquely positioned to act on that burden, provided the right competencies, indicators and governance structures are in place.

### 4.1. Environmental Impact of Hospitals and the Case for Sustainable Nursing

Hospitals generate large quantities of hazardous waste—biological, chemical and pharmaceutical—that can contaminate air, soil and water if not adequately managed [11,32]. Incineration, still the most common practice, releases dioxins and heavy metals such as mercury, contributing significantly to atmospheric pollution, while deficient waste management in settings with inadequate infrastructure increases the risk of infectious disease and toxic exposure for both staff and the population [11]. From a global perspective, the health sector generates between 1% and 5% of total pollution and is responsible for greenhouse-gas emissions, water pollution and excessive consumption of natural resources [1]; this footprint is compounded by intensive energy use for climate control, lighting and equipment sterilisation. Within this context, optimising the time and resources used in hospital units reduces waste and improves operational efficiency, contributing to hospital sustainability [23]. Strategies such as the Spanish PESMA establish intervention lines—waste reduction, optimised energy consumption and the promotion of ecological practices—to minimise the environmental impact of the sector without compromising care quality [36].

### 4.2. Energy Efficiency and the Limits of Nurse Sensitivity

The evidence indicates that energy efficiency is a fundamental pillar of a sustainable health system because it reduces operating costs, minimises environmental impact and improves working conditions. However, the NSEI framework [28] makes clear that energy and water consumption are only minimally sensitive to nursing action, in contrast to waste, which is highly nurse-sensitive. This distinction is methodologically important: it suggests that nursing-led interventions should prioritise the domains in which the profession has genuine leverage (segregation, reuse, rational use of materials, paper reduction and digitalisation) while contributing to energy and infrastructure strategies that are primarily the responsibility of management and facilities teams [16,20]. Digitalisation of clinical and administrative processes—electronic records, reduced paper use and virtual consultations—offers a clear example of a measure that simultaneously reduces energy demand and supports nursing workflows [12].

### 4.3. Waste Management as the Core Nurse-Sensitive Domain

Across the included studies, waste management consistently emerges as the domain where nursing can have the greatest measurable effect. Food waste alone may reach 30% of meals served [14], and operating rooms generate up to 30% of hospital waste because of their reliance on disposable materials [18]. Yet greener alternatives such as reuse and recycling remain underused owing to economic and logistical barriers [11]. The supply-chain perspective—twelve categories of practice capable of greening hospitals [9]—and integrative management approaches that combine environmental, social and economic measures [12] indicate that waste reduction is most effective when embedded in organisational systems rather than left to individual goodwill. Structured methods for choosing between single-use and reusable materials [24] and environmental-management systems such as ISO 14001 [22] provide practical, transferable templates for this purpose. At the scale of a single unit, replacing disposable pads with reusable linen eliminated roughly 496 kg of waste per year without increasing pressure-injury rates [26], and a retrospective ICU evaluation quantified the plastic-waste burden of single-use options across six respiratory procedures, identifying concrete scope for nurse-led substitution [25].

### 4.4. Carbon Footprint and the Value of Nursing Time

The carbon footprint of nursing activity is closely tied to how nursing time and resources are organised. The finding that 41.6–55.2% of nursing time is absorbed by unclassified or non-essential activities [23] points to a substantial efficiency reserve: restructuring nursing functions to reduce low-value activity would simultaneously improve care, staff well-being and environmental performance. Framing environmental factors as a clinical risk—responsible for a large share of global mortality [17]—reinforces the relevance of this agenda to everyday practice rather than treating sustainability as a peripheral concern.

### 4.5. Ecological Materials and Life-Cycle Thinking

The transition from disposable to reusable materials requires life-cycle thinking rather than simple substitution. The method proposed by Vanderwee et al. [24]—prioritising items by cost, consumption and reusability and then evaluating environmental impact, safety, cost-effectiveness and efficiency—offers a rigorous decision framework, and unit-level pathways such as the Green ICU [19] demonstrate how this thinking can be operationalised in resource-intensive settings. These approaches consistently place nursing education and engagement at the centre of successful implementation.

### 4.6. Social Value, Governance and the Move Toward Regeneration

The socio-health value of sustainable nursing is mediated by awareness, training and governance. Recurrent reports of limited climate awareness among staff [15,34] converge with governance analyses identifying knowledge, management involvement, professional commitment and technology use as the decisive—and currently limiting—factors for implementation [13]. The historical perspective [21,35] situates this agenda within the disciplinary identity of nursing, from Nightingale onward, rather than as an external imposition. Programmes such as MAS+ [37], operating within the Global Green and Healthy Hospitals framework [4] and aligned with national net-zero strategies [5], show that measurable emission reductions are achievable—in the MAS+ case, a 14.07% reduction in total institutional emissions, together with a 16.4% reduction in paper use and a 13.5% cut in emissions associated with inhalers and anaesthetic gases—when sustainability is adopted as a strategic priority with active staff participation—an early, concrete step toward the regenerative hospital model that motivates this review. This must nonetheless be read against the volume of evidence retrieved: only one included study explicitly addresses the regenerative transition, and it focuses on governance rather than on regeneration proper [13]. The shift from sustainability to regeneration announced in the title should therefore be understood as the conceptual horizon that frames this review, not as an evidenced pathway; what the mapped literature currently documents is a still-limited and predominantly descriptive body of evidence on nursing engagement in pro-environmental practice, strongest in waste management and education and weakest precisely where regeneration would begin—infrastructure, energy systems and organisational design.

### 4.7. Limitations

This review has several limitations. First, in line with the scoping-review approach, the evidence was mapped and charted descriptively rather than meta-analysed, and the narrative synthesis is more exposed to interpretive bias; because the included sources were not formally appraised for methodological quality as a criterion for inclusion, the review characterises the nature and extent of the evidence rather than grading its strength. In practical terms, this means that individual findings cannot be weighted by validated risk-of-bias judgements; the evidence-typology approach adopted (Section 2.5 and Section 3.1) mitigates—but does not replace—a formal appraisal with design-specific instruments such as the JBI critical-appraisal tools, and findings supported only by descriptive or institutional sources should therefore be regarded as provisional until confirmed by comparative primary research. Second, the review protocol was registered retrospectively rather than a priori, which may increase the risk of bias and limits protocol-based accountability, although the full protocol, the two-reviewer screening and charting process, and the search dates are reported transparently, and all post-registration changes (team expansion, clarification of the geographic scope and alignment of the search strategies, and the ScienceDirect field adaptation) are itemised in Section 2.1 and in the editable OSF component. The substantive consequence is that readers cannot verify that the research questions, criteria and synthesis plan preceded knowledge of the results, so the safeguard against selective reporting that a priori registration provides is weakened; the available mitigations—dual independent screening, dated search records and the immutable registration snapshot—reduce but do not eliminate this risk, and the removal of the geographic search block, although documented and compensated at the screening stage (Section 2.2), is itself a marker of imperfect a priori calibration of the protocol. The conclusions should therefore be read with this caution. Third, the restriction to publications in English and Spanish from 2021 to 2026 may have excluded relevant evidence in other languages or time periods. The temporal window was defined on scientific rather than merely pragmatic grounds—to isolate practice implemented after the 2019–2020 policy inflexion marked by sector-wide footprint quantification [2] and national net-zero strategies [5], and because earlier syntheses already cover the preceding period [9,10]—but it necessarily omits the foundational literature except as contextual references, and the language restriction, aligned with the languages the review team could appraise critically and with the Ibero-American coverage of SciELO and Dialnet, may have introduced a selection bias whose direction cannot be determined. Fourth, although the search was run without geographic restriction, the initial protocol wording referred preferentially to the European context and the final corpus is geographically heterogeneous (seven European sources, eight from other specific regions and ten global or multi-country sources), so the findings should be read as an international map with particular attention to European transferability rather than as a globally representative or exclusively European sample. Fifth, part of the evidence derives from grey literature and institutional reports of variable methodological transparency, and a few sources required careful appraisal of their indexing and peer-review status before inclusion. Finally, most included studies are descriptive or qualitative and report context-specific experiences, which limits the generalisability of the quantitative figures cited (e.g., percentage reductions in emissions or waste) and underlines the need for primary, comparative research using harmonised nurse-sensitive environmental indicators. In addition, the transition from sustainability to regeneration that gives this review its title is supported by a single governance-focused study [13]; the regenerative dimension of the evidence base is therefore incipient, and the title should be read as a conceptual framing of the direction of the field rather than as a description of the volume of regenerative evidence retrieved.

## 5. Conclusions

Hospitals are major contributors to the environmental footprint of the health sector, and nursing—as the largest professional group and the one closest to patients and clinical resources—is decisive in the transition toward green and, ultimately, regenerative hospitals. The synthesised evidence indicates that waste management is the domain most sensitive to nursing action—food waste alone may reach around 30% of meals served [14], operating rooms generate up to 30% of hospital waste [18], and a single-unit substitution of reusable linen for disposable pads eliminated roughly 496 kg of waste per year [26]—whereas energy and water use depend more on infrastructure and management decisions [28]. Institutional programmes that embed sustainability as a strategic priority have achieved reductions in the order of 14.07% in total emissions [37]. These figures are context-specific estimates rather than pooled effects, but they indicate the order of magnitude at stake. Education, leadership and the active engagement of nurses are the enablers most consistently reported, while insufficient training, resistance to change and limited investment are the recurrent barriers. Embedding environmental-sustainability competencies in nursing curricula and in hospital governance, and adopting nurse-sensitive environmental indicators to measure and benchmark progress, are therefore key levers for reducing the ecological footprint of clinical care without compromising its quality. The evidence supporting a genuinely regenerative model nonetheless remains incipient—confined in this review to a single governance-focused study—and much of the mapped literature is descriptive. Future research should therefore prioritise primary, comparative studies that quantify the impact of nurse-led interventions using harmonised indicators, so that the move from sustainability to regeneration can be grounded in robust, reproducible evidence.

## Figures and Tables

**Table 1:** Sources consulted and search strategies employed.

Database/Source	Search Terms (DeCS, MeSH, Free Text)	Boolean Operators
PubMed	Environmental exposure; Adverse effects; Sustainable development; Hospital administration; Sustainability; Nursing; Sustainable resource management; Carbon footprint	AND
Scopus	Nurses; Hospitals; Sustainable development; Green hospitals; Energy efficiency; Waste management; Eco-friendly materials; Carbon footprint; Regenerative hospitals (TITLE-ABS-KEY)	AND, OR
ScienceDirect	Sustainability; Hospitals; Nursing; Environment; Green hospitals; Eco-friendly hospitals; Environmental impact; Nursing care; Health-care waste management; Climate change; Energy efficiency	AND, OR
SpringerLink	Sustainability in hospitals; Nursing; Nurses; Nursing care; Climate change; Environmental impact of hospitals; Nursing practices; Hospital waste management; Sustainable healthcare	AND, OR, NOT
SciELO	Hospital sustainability; Nursing; Nursing practices; Environment; Sustainable development; Hospital waste	AND, OR, NOT
Dialnet	Sostenibilidad hospitalaria; Enfermería; Medioambiente; Hospitales verdes; Impacto ambiental; Gestión hospitalaria; Residuos hospitalarios; Cambio climático	AND, OR, NOT
UAM library catalogue	Hospital sustainability; Nursing; Waste management; Nursing practices; Environment	AND, OR
Institutional grey literature (other-methods arm)	WHO environmental health; Spanish Ministry of Health (PESMA); Health Care Without Harm; NHS England net-zero report; Fundación Jiménez Díaz (MAS+ programme)	—

Source: own elaboration. DeCS, Descriptors in Health Sciences; MAS+, Medio Ambiente y Salud (Environment and Health) programme; MeSH, Medical Subject Headings; NHS, National Health Service; PESMA, Plan Estratégico de Salud y Medio Ambiente (Strategic Plan for Health and the Environment); UAM, Universidad Autónoma de Madrid; WHO, World Health Organization. The UAM library catalogue and institutional grey literature are retrieval routes for the other-methods arm and are not counted as bibliographic-database records.

## Data Availability

No new data were created or analysed in this study. Data sharing is not applicable to this article.

## Supplementary Materials

The following supporting information can be downloaded at: https://www.mdpi.com/article/10.3390/healthcare14172701/s1, Supplementary Material S1: Search strategy and results by database (full search strings, database-specific adaptations and protocol deviations) [38]; Table S1: Sources, limits and records retrieved; Table S2: Full search strings (as run); Table S3: Dialnet queries (simple-search mode; 2021–2026; journal articles); Supplementary Material S2: PRISMA-ScR checklist [39].

## Abbreviations

The following abbreviations are used in this manuscript:

CINAHL	Cumulative Index to Nursing and Allied Health Literature
DeCS	Descriptors in Health Sciences
ICU	Intensive Care Unit
ISO	International Organization for Standardization
JBI	Joanna Briggs Institute
MAS+	Medio Ambiente y Salud (Environment and Health programme)
MeSH	Medical Subject Headings
NHS	National Health Service
NSEI	Nurse-Sensitive Environmental Indicator
OSF	Open Science Framework
PCC	Population–Concept–Context
PESMA	Plan Estratégico de Salud y Medio Ambiente
PRESS	Peer Review of Electronic Search Strategies
PRISMA	Preferred Reporting Items for Systematic Reviews and Meta-Analyses
PRISMA-ScR	Preferred Reporting Items for Systematic Reviews and Meta-Analyses extension for Scoping Reviews
UAM	Universidad Autónoma de Madrid
WHO	World Health Organization
