# Supplementary material for: From Sustainability to Regeneration: A Scoping Review of Nursing Practices in the Construction of Green and Healthy Hospitals
Source: Healthcare (Basel). 2026 Aug 24;14(17):2701. doi: 10.3390/healthcare14172701 (PMC13565624; doi:10.3390/healthcare14172701)
Supplement: Supplementary file 1 [file healthcare-14-02701-s001.zip › PRISMA-ScR_checklist.pdf]

PRISMA-ScR Checklist

Preferred Reporting Items for Systematic reviews and Meta-Analyses extension for Scoping Reviews

Manuscript: *From Sustainability to Regeneration: A Scoping Review of Nursing Practices in the Construction of Green and Healthy Hospitals*

Reference: Tricco AC, Lillie E, Zarin W, et al. PRISMA-ScR: Checklist and Explanation. Ann Intern Med. 2018;169(7):467–473.

#	Checklist item	Reported	Location in manuscript
**TITLE**			
1	**Title.** *Identify the report as a scoping review.*	Reported	Title page — title states "A Scoping Review".
**ABSTRACT**			
2	**Structured summary.** *Provide a structured summary (background, objectives, eligibility criteria, sources of evidence, charting methods, results, conclusions).*	Reported	Abstract (Background / Methods / Results / Conclusions).
**INTRODUCTION**			
3	**Rationale.** *Describe the rationale for the review in the context of what is already known.*	Reported	Section 1 (Introduction).
4	**Objectives.** *Provide an explicit statement of the questions and objectives.*	Reported	Section 1 (Introduction) — review aim and questions.
**METHODS**			
5	**Protocol and registration.** *Indicate whether a protocol exists and where it can be accessed; provide registration information.*	Reported	Section 2.1 — OSF registration (DOI 10.17605/OSF.IO/36KAG).
6	**Eligibility criteria.** *Specify characteristics of sources of evidence used as eligibility criteria (years, language, etc.) and rationale.*	Reported	Section 2 — eligibility criteria (2021–2026; English/Spanish; hospital nursing practice; transferable good practice).
7	**Information sources.** *Describe all information sources and the date of the most recent search.*	Reported	Section 2.2 and Supplementary Material S1 — six databases + grey literature; last search 28 May 2026.
8	**Search.** *Present the full search strategy for at least one database, including limits used.*	Reported	Section 2.2 and Supplementary Material S1 (Tables S1–S3) — full strings, MCL Calibrator, PRESS audit.
9	**Selection of sources of evidence.** *State the process for selecting sources (i.e., screening and eligibility).*	Reported	Section 2.2 / Section 3.1 — independent, duplicate screening against predefined criteria.
10	**Data charting process.** *Describe the methods of charting data and any processes for obtaining/confirming data.*	Reported	Section 2.5 — charting template; charted by one reviewer and verified by a second.
11	**Data items.** *List and define all variables for which data were sought and any assumptions/simplifications.*	Reported	Section 2.5 — reference, objective, design, setting/population, key findings.
12	**Critical appraisal of individual sources of evidence.** *If done, provide a rationale; describe the methods*	Reported	Section 2.5 — CASPe applied a posteriori for description only; never used to exclude studies (consistent with scoping-review

	and how the information was used.		methodology).
13	**Synthesis of results.** *Describe the methods of handling and summarising the charted data.*	**Reported**	Section 2.5 — descriptive narrative analysis; grouping into six thematic categories.
**RESULTS**			
14	**Selection of sources of evidence.** *Give numbers of sources screened, assessed for eligibility and included, with reasons for exclusion (flow diagram).*	**Reported**	Section 3.1 and Figure 1 — two-arm PRISMA-ScR flow (4,199 identified → 25 included).
15	**Characteristics of sources of evidence.** *For each source, present characteristics for which data were charted.*	**Reported**	Section 3 and Table 2 — design, setting/population, key findings for all 25 sources.
16	**Critical appraisal within sources of evidence.** *If done, present data on critical appraisal.*	**Reported**	Section 2.5 / Section 3 — CASPe ratings reported descriptively.
17	**Results of individual sources of evidence.** *For each included source, present the relevant data that were charted.*	**Reported**	Table 2.
18	**Synthesis of results.** *Summarise and/or present the charting results.*	**Reported**	Sections 3.2–3.7 and Figure 2 (thematic distribution).
**DISCUSSION**			
19	**Summary of evidence.** *Summarise the main results (including an overview of concepts, themes and types of evidence), link to objectives.*	**Reported**	Section 4 (Discussion).
20	**Limitations.** *Discuss the limitations of the scoping review process.*	**Reported**	Section 4 (Discussion) — limitations.
21	**Conclusions.** *Provide a general interpretation of the results with respect to the objectives, as well as potential implications.*	**Reported**	Section 5 (Conclusions).
**FUNDING**			
22	**Funding.** *Describe sources of funding and the role of funders for the review.*	**Reported**	Back matter — Funding statement.

All 22 PRISMA-ScR items are reported. Section numbers are stable; page numbers may shift on typesetting.
