# Supplementary material for: From Sustainability to Regeneration: A Scoping Review of Nursing Practices in the Construction of Green and Healthy Hospitals
Source: Healthcare (Basel). 2026 Aug 24;14(17):2701. doi: 10.3390/healthcare14172701 (PMC13565624; doi:10.3390/healthcare14172701)
Supplement: Supplementary file 1 [file healthcare-14-02701-s001.zip › Supplementary_S1_search_strategy.pdf]

Supplementary Material S1. Search strategy and results by database

Review: *From Sustainability to Regeneration: A Scoping Review of Nursing Practices in the Construction of Green and Healthy Hospitals*

Reporting standard: PRISMA-S (search reporting extension) / PRISMA-ScR.

Date of last search: 28 May 2026.

Language limits: English and Spanish.   Date limits: 2021–2026.

Search-strategy tool: Strings were developed, calibrated and translated using the MCL Search Calibrator (Mnemonics, Concepts and Links), v1.0.0 (Neves et al., 2026; https://doi.org/10.5281/zenodo.20423936; MIT licence), which maps concepts to controlled vocabularies (MeSH and Emtree), generates database-specific syntax for PubMed, CINAHL and Scopus, and audits the strategy following the PRESS checklist. For databases beyond PubMed, CINAHL and Scopus, the strings were adapted manually to their own syntax and limitations.

Table S1. Sources, limits and records retrieved

#	Database / source	Interface	Limits applied	Records
1	PubMed/MEDLINE	NCBI	2021–2026; English, Spanish	1,245
2	Scopus	Elsevier	2021–2026; English, Spanish; TITLE-ABS-KEY	2,058
3	ScienceDirect	Elsevier	2021–2026; Research + Review; Title–abstract–keywords	366
4	SpringerLink	Springer Nature	2021–2026; Article; Medicine and Public Health	417
5	SciELO	SciELO	2021–2026; Spanish/English	49
6	Dialnet	Dialnet	2021–2026; journal articles	64 †
	**Total identified (before cross-database de-duplication)**			**4,199**

† Dialnet: four term combinations returned 87 records gross (4 + 7 + 45 + 31); 64 unique after de-duplication (Table S3). The geographic restriction was removed from the PubMed and Scopus strings (protocol deviation; see below). These per-source counts correspond to the PRISMA-ScR flow diagram (Figure 1) and Section 3.1.

Protocol deviation (geographic limiter). *An earlier version of the PubMed and Scopus strategies included a block restricting results to Europe. This block was removed because it conflicted with the eligibility criteria, which expressly admit transferable examples of good practice from other regions; the strings below are reported as run, without geographic restriction.*

Table S2. Full search strings (as run)

1. PubMed/MEDLINE (NCBI)

```
("Nurses"[Mesh] OR nurs*[tiab] OR "nursing staff"[tiab] OR "nursing personnel"[tiab]) AND ("Hospitals"[Mesh] OR hospital*[tiab] OR "health facilit*"[tiab] OR "healthcare facilit*"[tiab] OR "medical cent*"[tiab]) AND ("Sustainable Development"[Mesh] OR "Conservation of Natural Resources"[Mesh] OR "Carbon Footprint"[Mesh] OR sustainab*[tiab] OR "green hospital*"[tiab] OR "green health*"[tiab] OR "energy efficien*"[tiab] OR "energy conservat*"[tiab] OR "waste management"[tiab] OR "medical waste"[tiab] OR "healthcare waste"[tiab] OR "eco-friendly material*"[tiab] OR "sustainable procurement"[tiab] OR "green material*"[tiab] OR "carbon footprint*"[tiab] OR "greenhouse gas*"[tiab] OR decarboni*[tiab] OR "net zero"[tiab] OR (regenerative[tiab] AND hospital*[tiab]) OR "regenerative health*"[tiab])
```

2. Scopus (Elsevier)

```
TITLE-ABS-KEY ( ( nurs* OR "nursing staff" OR "nursing personnel" ) AND ( hospital* OR "health facilit*" OR "healthcare facilit*" OR "medical cent*" ) AND ( "sustainable development" OR "conservation of natural resources" OR sustainab* OR "green hospital*" OR "green health*" OR "energy efficien*" OR "energy conservat*" OR "waste management" OR "medical waste" OR "healthcare waste" OR "eco-friendly material*" OR "sustainable
```

```
procurement" OR "green material*" OR "carbon footprint*" OR "greenhouse gas*" OR decarboni* OR "net zero" OR
( regenerative AND hospital* ) OR "regenerative health*" ) )
```

3. ScienceDirect (Elsevier) — field: Title, abstract, keywords

```
nursing AND hospital AND (sustainable OR sustainability OR "green hospital" OR "carbon footprint" OR "waste
management" OR "energy efficiency")
```

*Note: ScienceDirect does not support the * wildcard and caps queries at eight Boolean operators; the string uses base word forms and no geographic block. Applied to the title–abstract–keywords field and captured across successive result pages (366 records). Document-type and date limits applied via the interface.*

4. SpringerLink (Springer Nature)

```
(nursing AND hospital) AND ("hospital sustainability" OR "green hospital" OR "sustainable healthcare" OR "carbon
footprint" OR "waste management" OR "energy efficiency")
```

Note: SpringerLink searches full text by default; results were restricted via side filters (Content type: Article; Discipline: Medicine and Public Health; Date: 2021–2026).

5. SciELO

```
(enfermería OR enfermagem OR nursing) AND (hospital* OR hospitalar*) AND (sostenib* OR sustentab* OR sustainab*
OR "hospital verde" OR "green hospital" OR "residuos hospitalarios" OR "gestión de residuos" OR "huella de
carbono" OR "carbon footprint" OR "eficiencia energética")
```

6. Dialnet — see Table S3 (simple-search mode).

Table S3. Dialnet queries (simple-search mode; 2021–2026; journal articles)

Query	Records
enfermería sostenibilidad hospital	45
enfermería "residuos hospitalarios"	7
enfermería "huella de carbono"	4
sostenibilidad hospitalaria enfermería	31
**Sum (gross, with internal overlap)**	**87**
**Unique after de-duplication**	**64**

Notes on database-specific adaptations (per PRISMA-S)

- The PubMed strategy is the reference strategy; other databases were adapted to their own syntax and limitations, so the strings are not identical across sources.
- PubMed and Scopus: geographic "Europe" block removed (protocol deviation); strings reported as run without geographic restriction.
- ScienceDirect: no truncation; maximum eight Boolean operators; title–abstract–keywords field; captured across result pages (366).
- SpringerLink: full-text default search; narrowed with content-type, discipline and date facets (417).
- Dialnet: no complex Boolean support; four simple queries; counts overlap and were de-duplicated (87 gross → 64 unique).
- Grey literature (not counted as database records): World Health Organization; Spanish Ministry of Health (PESMA); Health Care Without Harm; NHS England net-zero report; Fundación Jiménez Díaz (MAS+ programme).

The per-source counts above match the PRISMA-ScR flow diagram (Figure 1) and Section 3.1: 4,199 records identified across the six databases; after de-duplication and screening, 25 studies were included (23 via databases and 2 via other methods).
